# Bibliographical

**Published:** 1867-12

**Authors:** 


### BIBLIOGRAPHICAL.

*Essentials of the Principles and Practice of Medicine, a Handy-Book for Students and Practitioners.* By HENRY HARTSHORNE, M. D., Professor of Hygiene in the University of Pennsylvania, Auxiliary Faculty of Medicine; formerly Professor of Practice of Medicine in the Medical Department of Pennsylvania College; lately Physician to the Episcopal Hospital of Philadelphia; Fellow of the College of Physicians of Philadelphia; Member of the American Philosophical Society, &c. &c. Philadelphia; Henry C. Lea, 1867.

We have here an octavo volume of 417 pages, which professes to give us the "essentials," *i. e.* what should at least be known by every medical man, of the principles and practice of medicine, and we must confess that the author has accomplished a confessedly difficult undertaking, as well perhaps as it was possible to do such a thing. That man who proposes to himself to condense within the limits of a work like this the immense subject of which it treats, except at the sacrifice of the first excellence of writing or speaking, viz: perspicuity, must be possessed of a faculty of condensation that falls to the lot of but few. Whatever is worth doing at all needs to be done well; a smattering knowledge of medicine is now the bane of the profession, and it is such knowledge that works like this necessarily impart. Our system of medical education, it is true, is subservient to, if not responsible for superficial professional attainments, and perhaps in this regard we are on an equal footing with the other so-called learned professions, all partaking of the nature and spirit of our young institutions, and influenced, to a great extent, by our peculiar political system. But these considerations are no apology for faulty books; there is no compulsion to authorship—the demand for books in every department of medicine is amply supplied. It may be that the work of our author is an improvement upon its predecessors in the same line; we confess to a want of information on this point, for we have entertained a life-long prejudice against labor-saving machines in the matter of knowledge—indeed we are incredulous as to there being any short or easy roads to its attainment. We have never consulted one of those learning-made-easy books, except on one or two occasions during the late war when some cramming was to be done, and Neil and Smith was the only thing in the shape of a book that was accessible. These strictures are not intended for this work alone—it has simply furnished an occasion for the expression of our views upon all works of its class. We can conceive of no utility in compends except as suggestive aids to students or others cramming for examination, and even for this purpose, the student must possess a a certain degree of familiarity with the subjects treated before he can derive any substantial benefit. For practitioners of medicine, we deem such works entirely unsuited—the subjects are so cursorily disposed of that we rise from their perusal dissatisfied and discontented, and thirsting for more thorough knowledge. Some physicians there be indeed, whose whole attainments consist of this kind of surface knowledge—who know a little of everything and nothing well—who require but a glance at a subject to satisfy their every want. For such we would recommend an epitome upon every subject.

As to the merits or demerits of the particular work before us we have this to say in advance: that there is no difficulty in ascertaining where the author stands (to use a familiar phrase) upon any subject he discusses—not a particle of ambiguity in the expression of his opinions. Indeed, there is a decided element of dogmatism running through the work, which is objectionable in a book which will be mainly used by tyros in medicine. There are many opinions and theories in medicine which are received and pass current as such for want of more accurate information; but at the same time, the learner should be informed that such opinions and doctrines may be accepted or rejected, as the advancing light of science may dictate. What is known in medicine should be carefully eliminated from what is unknown or conjectural, and a knowledge of their respective limits is the first substantial step in medical knowledge. We *know*, for example, an abortive treatment for malarial fever —we do *not know* such a treatment for typhoid fever, and the latter knowledge is just as important as the former. But a condensity of statement was essential to the plan of the work, and we, therefore, acquit the author of the charge of dogmatism. Indeed, we are free to confess that, in our humble opinion, a compend—a condensed or abridged statement of any subject or science—is the most difficult task of authorship, and requires talent of a rare and peculiar order. We feel satisfied that, had our author employed his capacity and experience in preparing a complete work on the principles and practice of medicine, he would have accomplished an undertaking that would have redounded far more to his credit and reputation than the work before us.

In regard to the grammar and style of the book, we must confess that they are not to our taste. Whether so or not, it bears the impress of having been prepared in a hurry.

There is a boldness and dash, a sort of reckless, don't-care spirit manifest in the style which we do not admire in a medical book. It reminds us somewhat of Prof. Meigs' style, in his celebrated letters to his class, "Woman and her Diseases," which received such reprobation from the medical press that the violation of good taste perpetrated in that work has not, to our knowledge, been repeated. Take an example: We open the book at page 97, and find the words "imperfection," "debilitation" and "desiderated" in one paragraph; in another place, "balancive" for balancing; and again: "I believe that a sound 'theory of medicine' may be expressed in a single paragraph, thus:—vital OPTIMISM is the aggregate tendency of all the forces of the living organism, under the controlling influence of *life-force*. But, the *best possible* result in a given case may, from its CONDITIONS and *circumstances*, fall far SHORT of *health*. Medicine, then, is to *favor or* SUPPLY *those conditions which, under natural laws, allow or promote the* BEST RESULT." There are innumerable evidences scattered through the book of this straining at effect on the part of the author, or of his fondness for using big words—*sesqui-pedalia verba*. There is a a want of ease or grace in his sentences—an awkward, bungling manner of expressing himself—that is painful to the reader; but still more censurable are the numerous and glaring grammatical errors with which the book abounds. Speaking of the treatment of inflammatory croup, we find, for example, the following: "Warm poultices, or cloths wrung out of cold water (which soon *becomes* warm when applied) may be applied to the throat." Now we are well aware that our author knows the difference between the singular and plural number, and that a plural nominative case must not be united with a singular verb. His work satisfies us that he is an educated man, and yet there is scarcely a page of it that does not exhibit a perfect contempt for the rules of grammar and good taste. Our author's views of pathology and treatment we regard objectionable on many points. We had hoped that the race of authors who advocate the indiscriminate use of the lancet, antimony and mercury, *et id omne genus*, in the treatment of acute disease, had either died out or passed into their serene and venerable dotage; that our eyes would not again behold a new work on the practice of medicine that sought to perpetuate these views. But we were mistaken. Dr. Hartshorne is a staunch believer in the old *regime;* he loves Bennett less than he does Rush; he accepts, indeed, and gives us a very full account of all the improved methods of treatment, but the new pathology, which ignores the so-called anti-phlogistic remedies, he will have none of. We cannot recommend your book, therefore, Doctor; we disagree with you *in toto* on these fundamental points; we would not have your book put into the hands of medical students (for whom it is intended) for the reason that it perpetuates what we believe to be grave errors.

And first on the subject of pneumonia, among its symptoms our author mentions delirium as of "*common*" occurrence. This, according to our observation, is not true: delirium does exist sometimes in pneumonia, but not often, and is of grave import. Hippocrates long ago recognized the serious prognostic significance of this symptom, and left us an aphorism on the subject: "*delirium in pneumonia est malum.*" But it is to his treatment of pneumonia that we desire to direct attention. "I am convinced," says Dr. H., "by experience, that prompt and moderate antiphlogistic treatment may greatly lesson the danger of pneumonia, if not shorten its duration. Probably five cases in six would recover without the abstraction of blood; the sixth might die for want of it. I believe that the mortality of pneumonia has increased in Philadelphia since blood-letting has been so generally abandoned. * * * * * * * Old persons, and those of feeble system, will neither bear nor require it." Now whether the mortality from pneumonia has increased or diminished in

Philadelphia in consequence of the general abandonment of venesection we know not; nor does this *ipse dixit* of our author relieve our doubts—a few facts and figures would have been much more satisfactory in the settlement of the question. An opinion like this, founded, perhaps, less upon facts than bias and prejudice in these days of statistics and accurate observation, passes like the idle wind, and is worth simply nothing in the adjudication of a great controverted question. In the above extract we have advocated the indiscriminate use of V. S. in all cases of pneumonia, except in the feeble and aged, for the reason that whilst five cases would recover without it, the sixth might die for want of it: that is to say, because one in six requires blood-letting, therefore all must be bled. Would it not be better to tell us how we should ascertain this sixth case that requires the lancet? to give us some rules or indications by which we could determine when to employ or omit this remedy? Fleishman, of Vienna, under the *nihilismus* treatment, lost but one in sixteen cases; Dietl, of the same city, experienced about the same result from putting his patients to bed and feeding them properly, and giving *no* medicine. Here was a fair test of the vain-glorious humbug, yclept homeopathy. Prof. Bennett, of Edinburgh, lost one in thirty-two and a quarter cases on the restorative treatment, the number treated being one hundred and twenty-five, running over a period of sixteen years' service in the Royal Infirmary of that city. Of these one hundred and twenty-five cases, twenty were complicated with different affections, and one hundred and five uncomplicated; of the latter not one died, although in fifteen of them the whole of one lung, and in twenty-six portions of both lungs, were involved.

From these data Prof. Bennett very properly concludes that "the first great fact which the preceding figures serve to establish is, that simple primary pneumonia, whether single or double, if treated by the restorative plan, is not a fatal disease. Surely one hundred and five cases, of which twenty-six were double, are sufficient to establish this proposition, especially when it is considered that they were diffused over sixteen years, and occurred in all seasons. Among these also the whole of one lung was involved in no less than fifteen cases, and the symptoms in many of them were exceedingly severe. Neither will anything as to the strength of constitution, or change of type, explain the result, as several of the cases were those of healthy, vigorous young laborers, whilst others were those of weak and broken-down seamstresses. In any and every case the disease goes through its natural progress, if the system be not too much exhausted, either naturally or by the interference of the physician; and if a judicious restorative treatment be adopted."

Although this is not the proper connection in which to give Prof. Bennett's views in full on the pathology and treatment of pneumonia, we connot refrain from presenting a brief outline of a treatment which gives us such remarkable results.

"For palliating symptoms, and especially pain and dyspnœa, warm fomentations and poultices I believe to be the best and safest remedies. Chloroform has been given by Varentrapp, and others, with good effect. No doubt small bleedings, to the extent of 8 or 10 ounces, give relief; but in debilitated persons they are dangerous, and in all tend, by weakening the strength at a period when the depressed system is struggling to gain its equilibrium, to prolong the convalescence and favor dangerous sequellæ. Still, a small bleeding may be employed as a palliative with caution, to relieve engorgement of the lungs, and congestion of the right side of the heart, although it is very rarely required. It should be remembered, in cases of double pneumonia, that there is often great dyspnœa on the sixth or seventh day, which will generally yield to warm poultices locally, and moderate doses of wine.

As a curative treatment, I am satisfied that the best plan is rest in bed, nutritive drinks, especially good beef tea, *from the first*, assisted by 4 to 8 oz. of port wine, if the pulse becomes weak, and solid nutrients as soon as they can be taken. The elimination of the exudation may be further assisted by salines, (Acetate of Ammonia, and small doses of Tartar Emetic, 1-16th of a grain) and diuretics (Nitric Ether), although nature will accomplish this herself if the strength of the body be maintained. All active purgatives, contra-stimulants, depressants, anodynes, and lowering remedies of every description, should be avoided."

Now, we would frankly ask the candid reader whether he loses but one in thirty-two and a quarter cases under his treatment of this hitherto considered dangerous disease? We are quite sure that the so-called antiphlogistic treatment has afforded no such favorable results in our hands, and hence have virtually abandoned it. Why is not this conservative treatment generally adopted? is not Bennett's veracity to be relied upon? he is not alone in these views—hundreds of others, the flower of the profession the world over, daily practice and teach a similar doctrine. From time immemorial, dogmatism and pride of opinion have been the great obstacles to the dissemination of truth. Who does not now believe that a do-nothing treatment of typhoid fever is better than the heroic course in vogue thirty years ago, which consigned so many to their graves? and yet it is well known that many physicians adhered to that course until actually driven from it by the public opinion of their patrons. We see, even to this day, in almost every community, specimens of this class of obsolete practitioners, who have been utterly abandoned by public patronage because of their adhesion to the old practice of puking, purging and bleeding. Now, is it not just possible that other acute diseases besides typhoid fever do not require—nay, even do not tolerate, active interference? We believe so, and could give our reasons for such belief, but this is not the place nor the time.

On the subject of "true croup," our author is equally unfortunate. "Pseudo-membranous, or true croup, does not generically differ from inflammatory croup; of which it is only a grade or termination; *i. e.* any case of inflammatory or catarrhal croup *may* end in the exudation of coagulable lymph within the air tubes. Whether this shall occur or not, in any given case, depends, *a*, on the degree of inflammation; *b*, on the state of the blood of the patient; *c*, on the treatment." If we understand the text, it is here taught that the presence of the membranous exudation depends upon the *degree* of the inflammation, and that its formation may be prevented by treatment. That is to say, that this peculiar fibrinous exudation may occur in *simple* acute laryngitis if the inflammation be intense in degree or not prevented by antiphlogistic treatment. This, in our opinion, is totally erroneous pathology, and tends to mischievous results in practice. The term croup is vaguely used in medicine: it is employed to express three distinct and dissimilar pathological conditions. 1. The spasmodic croup, the essential pathological element of which is spasm of the laryngeal muscles without inflammation or exudation of any kind. Spasm of the glottis would be a better name for this condition than croup. 2. Acute simple laryngitis, which gives rise to the croupal symptoms of hoarse, ringing cough, and loud, noisy breathing—the croupal phenomena in this disease depend mainly on spasm of the laryngeal muscles. 3. Exudative laryngitis, or true membranous croup, *i. e.* a specific inflammation of the larynx, attended *ab initio* with fibrinous exudation upon the laryngeal mucous membrane. Now, in regard to the second form, no matter how intense the inflammation, we hold that it is never attended with the plastic exudation which constitutes the pseudo-membrane of true croup. The symptomatic fever is always higher in this form of laryngeal inflammation than in true croup, so that if the membranous exudation depended upon the *degree* of inflammatory action, we would generally have it in acute simple laryngitis. Whilst plastic exudation is not one of the pathological elements of this disease, it is nevertheless a serious condition. There may be thickening of the mucous membrane and sub-mucous infiltration to such an extent as to seriously obstruct the rima glottidis, and thus, as happens in true croup, endanger life mechanically. It is here that the anti-phlogistic treatment —if indeed there be any combination of remedies worthy of that appellation—is imperatively demanded. The inflammation must be *jugulated*, if possible; and it is here that we would faithfully employ our author's treatment for true croup. It must be admitted, however, that a vigorous anti-phlogistic treatment is consistent with his pathology of croup—if it be due to the intensity of the inflammation, the rational object of treatment is to subdue it. But it does not depend on the severity of the local phlegmasia, but on "an underlying, special, constitutional, morbid condition." What, then, are the objects of treatment in true croup? The plastic exudation takes place early—generally before the patient is seen by the physician—no measures then need be resorted to with the hope of *preventing* it. What becomes of the exudation? It is exfoliated by a suppurative process taking place between it and the subjacent mucous membrane. The rational object of treatment, then, is to promote this vital act of exfoliation, which depends upon the strength and vigor of the system, and which will certainly take place in due course of time, if the patient is not previously cut off by mechanical obstruction of the glottis.

Does our author's anti-phlogistic treatment promote this vital act of seperation? We believe not; and further, that the great mortality of the disease is due to a mischievous treatment, based upon an erroneous pathology. Vomiting and purging, leeching and bleeding, calomel and antimony, are not well calculated to preserve the vital energies in a disease of self-limited duration, and the result of such practice does not tend to encourage its continuance.

We will conclude our notice of the work before us by a few observations on the author's treatment of remittent fever. "As soon as the violence of systemic excitement has been moderated," (and this is all italicized) by purgatives and blood-letting, we *may* begin with quinine, but unless some malignancy is suspected, a single grain every two hours, or, at most, but a grain every hour, will be sufficient. Need we tell our Southern readers that this is all nonsense! Suppose the remission lasts but a few hours—three, or four, or five, or six—how much quinine could be taken before the accession of the next paroxysm? Why, five or six grains, at most. Ah, Doctor, we can tell you, our overseers down here can do better than that with malarial fevers.

Notwithstanding our objections to the "essentials"—and we regret that there should have been any—it contains a vast amount of useful information, compressed and condensed into a small compass, and is verily *multum in parvo*.

D. C. O'K.

